# Nutritional status among young adolescents attending primary school in Tanzania: contributions of mid-upper arm circumference (MUAC) for adolescent assessment

**DOI:** 10.1186/s12889-019-7897-4

**Published:** 2019-11-27

**Authors:** Margaret Lillie, Isaac Lema, Sylvia Kaaya, Dori Steinberg, Joy Noel Baumgartner

**Affiliations:** 1 Duke Global Health Institute, Duke University, Durham, USA; 2 School of Medicine, Muhimbili University of Health & Allied Sciences (MUHAS), Dar es Salaam, Tanzania; 3 School of Nursing, Duke University, Durham, USA

**Keywords:** Tanzania, Mid-upper arm circumference, Adolescent nutrition, Assessment of nutritional status, Body mass index, Anthropometry

## Abstract

**Background:**

Adolescence is a critical time of development and nutritional status in adolescence influences both current and future adult health outcomes. However, data on adolescent nutritional status is limited in low-resource settings. Mid-upper arm circumference (MUAC) has the potential to offer a simple, low-resource alternative or supplement to body mass index (BMI) in assessing nutrition in adolescent populations.

**Methods:**

This is secondary data analysis, from a cross-sectional pilot study, which analyses anthropometric data from a sample of young adolescents attending their last year of primary school in Pwani Region and Dar es Salaam Region, Tanzania (*n* = 154; 92 girls & 62 boys; mean age 13.2 years).

**Results:**

The majority of adolescents (75%) were of normal nutritional status defined by BMI. Significantly more males were stunted than females, while significantly more females were overweight than males. Among those identified as outside the normal nutrition ranges, there was inconsistency between MUAC and BMI cut-offs. Bivariate analyses indicate that BMI and MUAC show a positive correlation for both female and male participants, and the relationship between BMI and MUAC was more strongly correlated among adolescent females.

**Conclusions:**

Further studies are needed with more nutritionally and demographically diverse populations to better understand the nutritional status of adolescents and the practical contribution of MUAC cut-offs to measure adolescent nutrition.

## Background

Apart from reproductive health risk assessments, comprehensive adolescent health assessments are frequently neglected in clinical encounters, research, and programming in low and middle income countries (LMIC) [1, 2]. Without quality health information systems inclusive of adolescent health data, stakeholders are working in a data vacuum. This is particularly problematic for assessing adolescent nutritional status [3–5]. Historically, undernutrition for children under age five has been the overwhelming nutritional priority in LMICs [6]. However, young adolescents in LMICs are also particularly at risk of undernutrition because they are often already stunted, have anaemia, and/or have existing infections from childhood [7]. For adolescent females, in particular, undernutrition contributes to a larger cycle of intergenerational undernutrition because adolescent mothers, and mothers who are undernourished from adolescence are more likely to have low birth weight infants which can lead to higher mortality rates, stunting, impaired mental development, and increase risk of chronic disease for their children [8–10]. While undernutrition continues to be a concern, with increasing globalization and development, LMICs are also experiencing an increase in overnutrition, creating a “dual burden” of under- and overnutrition [8].

Anthropometric measurements and their corresponding referent curves are positioned as important indicators for assessing nutritional status for adolescents due to their comparability across sex and age [11–13]. Age adjusted growth curves for body mass index (BMI) and height for children ages 5–19 were produced by the World Health Organization a decade ago [14]. In addition to height-for-age and BMI-for-age, mid-upper arm circumference (MUAC), or the measurement of the circumference of the arm halfway between the tip of the elbow and the tip of the shoulder, has the potential to offer a simple alternative or supplement to BMI in assessing nutrition in adolescent populations. The BMI measurement can be affected by factors that mask overall body mass, such as trunk oedema due to protein-energy malnutrition. MUAC is unaffected by this symptom [15]. BMI also requires adequate equipment, namely a standometer and functioning scale. In contrast, MUAC has the advantages of simple equipment (a standardized tape in millimetres created for MUAC measurements) and can be used by clinicians and non-clinicians alike in clinical and non-clinical settings, allowing for more efficiency in clinical practice and programs in low-resource settings [12, 16, 17].

Standardized cut-offs for MUAC in adolescent populations have not been established due to lack of studies and country level data on MUAC measurements in adolescents [17, 18]. While there is an emerging body of evidence that MUAC and BMI correlate in adolescent populations, there are numerous calls for more evidence of this correlation for different age groups and geographic locations [17–23]. In support of broader use of MUAC, in 2017, Mramba et al. published MUAC-for-age growth curves for 5–19 year old children. These MUAC-for-age growth curves were created to be used with the WHO BMI-for-age growth curves for assessing undernutrition using the same data sets that were used to construct WHO reference curves for adolescents. These curves were validated using data from Kenya, Zimbabwe, and Uganda and deemed as effective as the WHO BMI-for-age cut-offs for assessing mortality risk due to undernutrition, however further research is needed to demonstrate the practical use of Mramba et al.’s findings [16, 24].

Understanding the nutritional status of adolescents is of particular importance in Tanzania. About one-quarter of the population of Tanzania are adolescents between 10 and 19 years old, yet the only national measures of adolescent nutrition are data from the 2015/2016 Demographic and Health Surveys (DHS) which reports anaemia and BMI measures for females 15 to 19 years old [25, 26]. The DHS reports that of Tanzanian females ages 15–19, 47% are anaemic, 18% have a less than normal BMI, and 10% have a greater than normal BMI [26]. Although helpful measures, there is a distinct lack of information on the nutritional status of younger females ages 5–15 and males 5 years and above. This country-level data gap on nutritional status of young adolescents in Tanzania limits the country’s ability to generate evidence for action to support programs that promote optimal health for adolescents during this critical period of growth [27].

While the Tanzania DHS does not broadly target adolescents, there are resources supported by the Tanzanian Ministry of Health, Community Development, Gender, Elderly and Children (MCDGC) that support nutrition assessment across the life course. The USAID supported Food and Nutrition Technical Assistance III Project (FANTA) and the MCDGC have partnered to not only create the Tanzania Food and Nutrition Center within the MCDGC but have also created the Tanzania Nutrition Assessment, Counseling, and Support (NACS) tools and trainings to assist providers in giving better nutrition care [27]. These tools support the WHO BMI-for-age z-scores to be used for children and adolescents 5–17 years old and offer cut-offs for MUAC for adolescents 10–14 year olds and 15–17 year olds. While these resources undoubtedly support a range of nutrition programming efforts, there is no reported data-driven evidence base to support these cut-offs [15].

The primary aims of this secondary analysis were 1) to describe the nutritional status of in-school adolescents in two Standard 7 primary school classes in Tanzania using BMI, height, and MUAC, 2) to compare the currently available, age-specific MUAC cut-offs to BMI cut-offs, and 3) to determine the relationship between MUAC and BMI as indicators of nutritional status stratified by sex.

## Methods

### Parent study/ethics

Data from this study come from a pilot study assessing the feasibility and acceptability of Adolescent Wellness Visits, a service provided via a collaboration between schools and nearby health facilities, to provide reproductive health and non-reproductive health services including assessments and information on nutrition, vision, dental health, mental health, puberty, contraception, and HIV testing and counselling [Duke University Center for AIDS Research (CFAR) Small Grant, NIH 5P30 AI064518]. The intervention targeted Standard 7 students because they are in the last year of primary school before many students drop out prior to moving on to secondary school [26]. All study procedures were approved by the ethical review boards at Duke University, Muhimbili University of Health and Allied Sciences, and the National Institute of Medical Research of Tanzania. Before conducting the wellness visits, nurses at the study health facilities were trained and provided time to practice all aspects of the wellness visit. For anthropometric measurements, clinicians were trained by government district nutrition officers on how to measure height and weight and how to use MUAC measuring tapes and BMI wheels. Training participants practiced on the same person multiple times in order to achieve interrater reliability. Training participants were also provided with a manual to refer to after the training. For MUAC measurements, the manual contained information from the NACS job aid and training manual [15, 28].

### Participants

All students currently enrolled in Standard 7 at the participating primary schools were eligible to participate in the study if they had given written assent themselves and had written consent from a parent/guardian. Of the 173 students in the two Standard 7 classes, 156 students were fully consented in the study and 154 completed the wellness visit.

### Setting

Data were collected from a government primary school and a matched government health dispensary in Dar es Salaam Region (urban) and a government primary school and matched government district hospital in Pwani Region (peri-urban). These settings were chosen due to the district level interest in piloting the Adolescent Wellness Visit model.

### Data collection

All participating adolescents were administered a survey after they completed their Adolescent Wellness Visit with the nurse at the health facility. The quantitative survey covered demographic characteristics and their experiences participating in the wellness visits. The assent and consent forms included permission for us to access their medical record data from the wellness visit encounter. Patient forms containing anthropometric measures were filled out by medical providers for each adolescent participant and entered electronically at the end of the survey by research assistants. Data was collected electronically on encrypted Android tablets through the Qualtrics Offline Surveys application Version 1.4 and uploaded to the Qualtrics server at the end of each day. Data collection instruments were translated into Kiswahili and administered by bilingual local research staff (recent MD graduates).

### Demographic measures

We collected data on age, household size, religion, parent mortality, parent occupation, food security, puberty and socio-economic status. Food security was measured through a self-reported indicator of food access during the previous 4 weeks. The question is from the Household Food Insecurity Access Scale (*In the past 4 weeks, did you ever worry that your household would not have enough food?*) [29]. Pubertal status was measured through self-reported measures. Females were asked whether they had started their menstrual period. Males were asked if they have noticed any pubertal changes such as voice changes or experiencing a wet dream.

### Anthropometric measures

Anthropometric measures were taken one time in a private room as part of the Adolescent Wellness Visit at the participating clinic. Height was measured in metres to the nearest centimetre with a clinic-provided stadiometer (light clothing, without shoes) as a continuous variable and transformed into height-for-age z-scores based on sex and age in months through the WHO STATA macro package based on the WHO height-for-age curves [30]. Height-for-age z-scores were categorized based on the WHO stunting cut-offs (Stunted: <− 2SD; Not stunted: ≥ − 2 SD) [31]. Weight was measured in kilograms to the nearest tenth of a kilogram with a clinic-provided beam scale. BMI scores used in the study were calculated from the provider reported height and weight variables (kg/m2) and transformed into BMI-for-age z-scores based on sex and age in months through the WHO STATA macro package based on the WHO BMI-for-age curves [31]. BMI-for-age z-scores were categorized based on the WHO BMI cut-offs for under and overnutrition (Obese: >2SD; Overweight: >1SD; Normal: 1 - -2SD; Thinness: <− 2 SD; Severe thinness: <− 3 SD) [32]. MUAC was measured in centimetres to the nearest millimetre as a continuous variable with standardized MUAC measuring tapes provided by the FANTA project. MUAC was measured on the bare arm of each participant halfway between the tip of the elbow and the tip of the shoulder, in accordance to the NACS job aid and manual. MUAC was transformed as a categorical variable using MUAC cut-offs for 10–14 years and 15 years and above recommended by NACS (Normal: 10–14 years old: ≥ 18.5 cm, 15–17 years old: ≥ 22.0 cm; Moderate Acute Malnutrition: 10–14 years old: 16.0–18.5 cm, 15–17 years old: 18.5–22.0 cm; Severe Acute Malnutrition: 10–14 years old: < 16.0 cm, 15–17 years old: < 18.5 cm) [15, 28]. MUAC was also transformed into a categorical variable using MUAC-for-age z-scores calculated and categorized using an *R Shiny* web tool provided through correspondence with Jay Berkley and Lazarus Mramba, authors of the MUAC-for-age growth curves (z < − 3, − 2 < z > − 3, and z > − 2) [24]. While not distinctly naming the categorized data, Mramba et al. found that children that had MUAC-for-age z-scores of − 2 to − 3 and less than − 3 was associated with hazard ratios for death within 1 year compared to z-scores of − 2 or more [24].

### Data analysis

For all continuous, bivariate analyses, a Wilcoxon rank sum test was used due to the non-parametric nature of the variables and the small sample size. For all categorical, bivariate analyses, a Fisher’s Exact test was used for all variables that had cells with values less than 5 and a ***χ***^2^ test was used for the remaining variables.

To determine the relationship between BMI calculations and MUAC measurements a Spearman’s rank correlation coefficient was calculated due to the non-parametric distribution of MUAC values [33].

To examine the relationship between BMI and MUAC cut-offs, sensitivity and specificity were calculated for the *NACS* (< 18.5 cm for 10–14 year olds; < 22.0 cm for 15–17 year olds) and Mramba et al. cut-offs (z < − 2) when compared to BMI-for-age z-score cut-offs (z < − 2). Here, sensitivity refers to the proportion of those who were undernourished based on age-specific” gold standard”, who were also undernourished based on the comparison cut off. Specificity, in this case, refers to the proportion of those who were not undernourished (over-nourished or normal) based on the “gold standard”, who were also not undernourished (normal) based on comparison cut-offs.

All analyses were conducted using the statistical software package STATA 15.

## Results

### Demographics

The socio-demographic characteristics of the study participants by study location are presented in Table 1. There were 61 female participants and 41 male participants in Dar es Salaam and 31 female participants and 21 male participants in Pwani. Pwani participants were on average a year older than those in Dar es Salaam. Relatedly, more students in the Standard 7 class had reached puberty in Pwani, than in Dar es Salaam. More participants in Dar es Salaam reported having tap water inside their home and more participants in Pwani reported having electricity inside their homes. Father occupation differed between the two regions; with fathers in Pwani mainly working sales or services/clerical jobs and agriculture or fishing and fathers in Dar es Salaam mainly working in skilled and unskilled manual labour.

**Table 1:** Socio-demographic characteristics of study sample (*n* = 154)

Variable	Total *n* = 154	Pwani *n* = 52	Dar es Salaam *n* = 102	*p*-value
Mean Age (range)	13.2 (11,18)	13.8 (12, 16)	12.9 (11, 18)	0.000
Mean # of people (including self) in household^a^ (range)	6.4 (3,25)	6.8 (3, 25)	6.3 (3,15)	0.596
		% (n)		
Religion				0.000
Muslim	63.6 (98)	92.3 (48)	49.0 (50)	
Christian	35.7 (55)	5.8 (3)	51.0 (52)	
Father Muslim/ Mother Christian	1 (0.6)	1 (1.9)	0 (0.0)	
Parent Mortality^b^				0.865
Both Parents Alive	88.9 (136)	92.3 (48)	87.1 (88)	
Mother Deceased	2.0 (3)	1.9 (1)	2.0 (2)	
Father Deceased	8.5 (13)	5.8 (3)	9.9 (10)	
Both Parents Deceased	0.7 (1)	0.0 (0)	1.0 (1)	
Mother’s Occupation^c^				.083
Professional/Technical/Managerial	2.0 (3)	0 (0.0)	3.1 (3)	
Clerical/ Sales & Services	30.9 (46)	41.2 (21)	25.5 (25)	
Manual (skilled or unskilled)	28.9 (43)	17.7 (9)	34.7 (34)	
Agriculture/Fishing	7.4 (11)	5.9 (3)	8.2 (8)	
Not currently working/Domestic Service	26.9 (40)	33.3 (17)	23.5 (23)	
Do not know	4.0 (6)	2.0 (1)	5.1 (5)	
Father’s Occupation^d^				0.000
Professional/Technical/Managerial	11.6 (16)	10.2 (5)	12.4 (11)	
Clerical/Sales & Services	13.8 (19)	24.5 (12)	8.9 (7)	
Manual (skilled or unskilled)	45.7 (63)	16.3 (8)	61.8 (55)	
Agriculture/Fishing	14.5 (20)	28.6 (14)	6.7 (6)	
Not currently working/Domestic Service	2 (1.5)	0.0 (0)	2.3 (2)	
Do not know	13.0 (18)	20.4 (10)	9.0 (8)	
Puberty				0.000
Started puberty	52.6 (81)	67.3 (35)	45.1 (46)	
Not started puberty	47.4 (73)	32.7 (17)	54.9 (56)	
Food Insecurity in Past 4 weeks				0.493
Yes	11.0 (17)	9.6 (5)	14.7 (15)	
No	89.0 (137)	90.4 (47)	85.3 (87)	
Equity Tool Items				
Tap water inside home				
Yes	56.5 (67)	25.0 (13)	47.1 (54)	0.001
No	43.5 (87)	75.0 (39)	52.9 (48)	
Toilet inside house or compound				0.274
Yes	94.2 (145)	98.0 (51)	92.2 (94)	
No	5.8 (9)	2.0 (1)	7.8 (8)	
Electricity inside house				0.006
Yes	66.2 (102)	81.8 (42)	58.8 (60)	
No	33.8 (52)	19.2 (10)	41.2 (42)	
Someone in household with mobile phone				
Yes	100.0 (154)	100.0 (52)	100.0 (102)	
No	0.0 (0)	0.0 (0)	0.0 (0)	
Television inside house				0.200
Yes	66.2 (102)	73.1 (38)	62.7 (64)	
No	33.8 (52)	26.9 (14)	37.3 (38)	
Main material of Floors				0.081
Earth, Sand, Dung	7.1 (11)	9.6 (5)	5.9 (6)	
Ceramic Tiles	24.0 (37)	13.5 (7)	29.4 (30)	
Cement	68.2 (105)	76.9 (40)	63.7 (65)	
Carpet	0.6 (1)	0.0 (0)	1.0 (1)	

^a^No major differences in means and *p*-value with or without outlier

^b^*n* = 153 ^c^*n* = 149 ^d^*n* = 138

### Anthropometric measures

Descriptive anthropometric measures categorized by sex are presented in Table 2 and summarized in the following sentences. Overall, females had statistically higher height, weight, BMI, and MUAC measurements than males. Although malnutrition indicated by cut-offs varied, up to 25% of the study population was outside of normal nutrition range. There were significant differences between males and females for height-for-age z-scores measuring stunting, BMI-for-age z-scores measuring under- and overnutrition, and undernutrition using NACS guidelines cut-offs. There was no significant difference between self-reported puberty attainment between males and females. Significantly more males than females were stunted according to height-for-age z-score cut-offs. Males and females had a significantly different distribution of under- and overnutrition according to BMI-for-age z-score cut-offs, with 17.4% of females being overweight compared to 4.8% of males and with 9.7% of females being underweight compared to 17.7% of males. A greater proportion of males were categorized as having moderate acute malnutrition according to the NACS guidelines (22.6%) than females (5.5%). The same trend is presented using the Mramba et al. z-score cut-offs with 19.3% of males in the <− 2 standard deviations category compared to 6.6% of females.

**Table 2:** Anthropometric Measures and Pubertal Status by Sex

Variable	Total *n* = 154	Males *n* = 62	Females *n* = 92	*p*-value
Mean Height (range)^a^	151.6 cm (130, 170.9)	149.5 cm (130, 170.9)	153.1 (134.4, 169.4)	0.014
Mean Weight (range)	42.2 kg (25, 72)	39.1 kg (26, 58.9)	44.2 kg (25, 72)	0.000
Mean BMI (range)	18.3 (12.8, 31.2)	17.4 (13.5, 30.3)	18.8 (12.8, 31.2)	0.002
Mean MUAC (range)	22.3 cm (16.5, 33)	21.4 cm (18, 33)	23.0 cm (16.5, 31.6)	0.000
		% (n)		
Pubertal Status				0.742
Started puberty	52.6 (81)	54.8 (34)	51.1 (47)	
Not started puberty	47.4 (73)	45.16 (28)	48.9 (45)	
Height-for-age z-score categorized (sex specific)				0.000
Not Stunted (≥ −2 SD)	85.7 (132)	69.3 (43)	96.7 (89)	
Stunted (<−2 SD)	14.2 (22)	30.6 (19)	3.2 (3)	
BMI-for-age z-scores categorized (sex specific)				0.047
obese (>2SD)	1.3 (3)	1.6 (1)	1.1 (1)	
overweight (> 1SD)	11.0 (17)	3.2 (2)	16.3 (15)	
normal (1 - -2 SD)	74.7 (115)	77.4 (48)	72.8 (67)	
thinness (<−2 SD)	10.4 (16)	14.5 (9)	7.6 (7)	
severe thinness (<−3 SD)	2.6 (4)	3.2 (2)	2.1 (2)	
MUAC-for-age z-scores categorized (Mramba et al.)				0.004
z > −2	88.2 (135)	80.6 (50)	93.4 (85)	
-2 < z > − 3	10.5 (16)	19.3 (12)	4.4 (4)	
z < −3	1.31 (2)	0.0 (0)	2.2 (0)	
MUAC categorized^a,b^ (not delineated by sex, age adjusted) *Per NACS guidelines*				0.002
Normal	87.0 (134)	77.4 (48)	94.5 (86)	
Moderate acute malnutrition (MAM)	12.3 (19)	22.6 (14)	5.5 (5)	

^a^*n* = 153

^b^0% had severe acute malnutrition (SAM)

### Relationship between BMI and MUAC

**Fig. 1:**
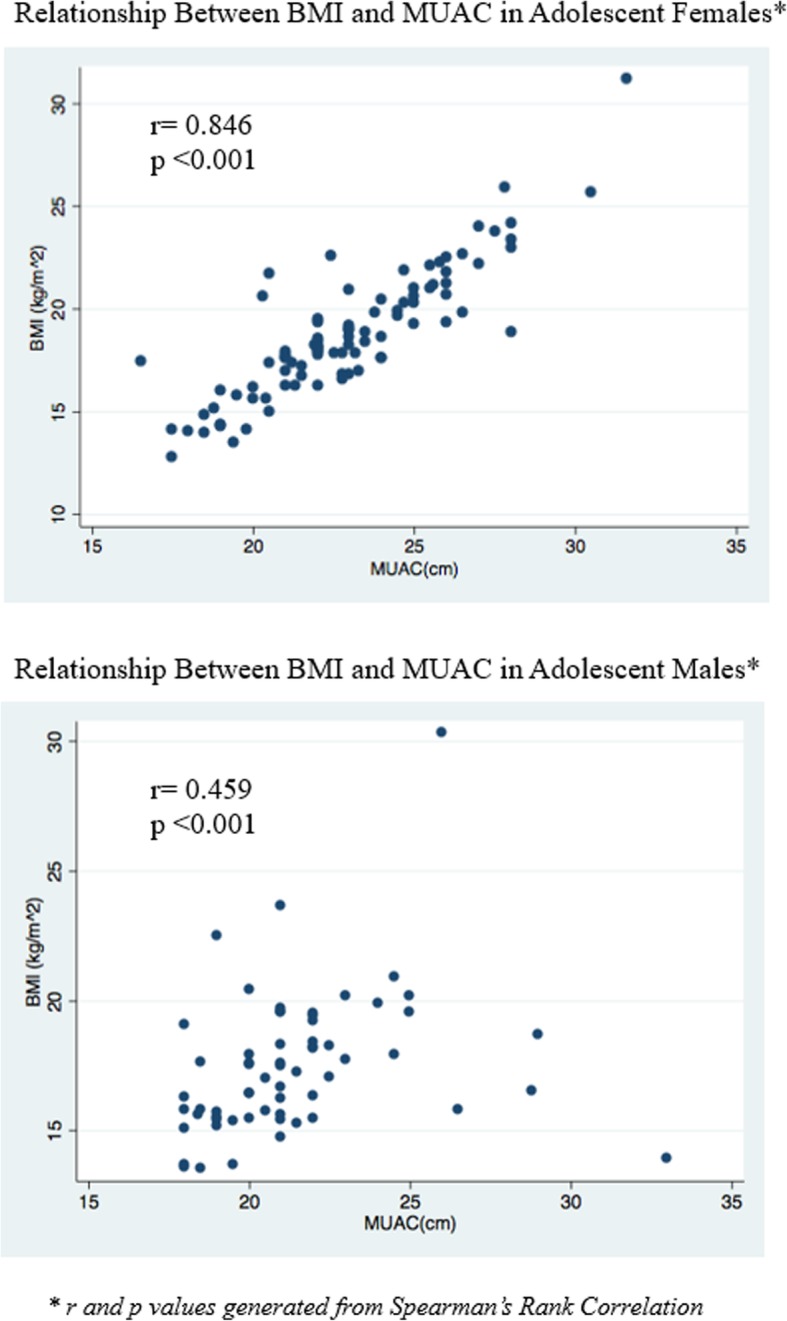
Relationship between BMI and MUAC Measurements in Adolescent Females and Males

Bivariate analyses indicate that BMI and MUAC measurements show a positive correlation for both female and male participants, though the relationship between BMI and MUAC was more strongly correlated among adolescent females (*R* = 0.846, *p* = < 0.001) compared to adolescent males (*R* = 0.459, *p*-value< 0.001) (Fig. 1).

Exploratory sub-group analyses based on the BMI-for-age cut-offs, performed to give an initial indication of direction and magnitude for future inquiry, revealed that in males and females who were undernourished, there was no correlation between BMI and MUAC (males: *n* = 11, *r* = 0.442, *p* = 0.173; females: *n* = 9, *r* = 0.246, *p* = 0.522). In females, there was a stronger correlation between BMI and MUAC in those who were overnourished (*n* = 16, *r* = 0.851, *p* = < 0.001; note: sample too small for boys, *n* = 3). In males, the correlation between BMI and MUAC remained significant after categorizing between those who were stunted (*r* = 0.535, *p* = 0.018) and those who were not stunted (*r* = 0.591, *p* < 0.001). In females, there was no difference in correlation between BMI and MUAC between those who had reported attaining their menstrual cycle (*r* = 0.858, *p* < 0.001) and those who had not (*r* = 0.803, =0.000. For males, there was a weak correlation between BMI and MUAC in participants who had reported signs of puberty (*r* = .5122, *p* = 0.002) and there was no correlation between BMI and MUAC for those who had not started puberty (*r* = 0.381, *p* = 0.052).

### Comparing available MUAC cut-offs and BMI cut-offs

**Fig. 2:**
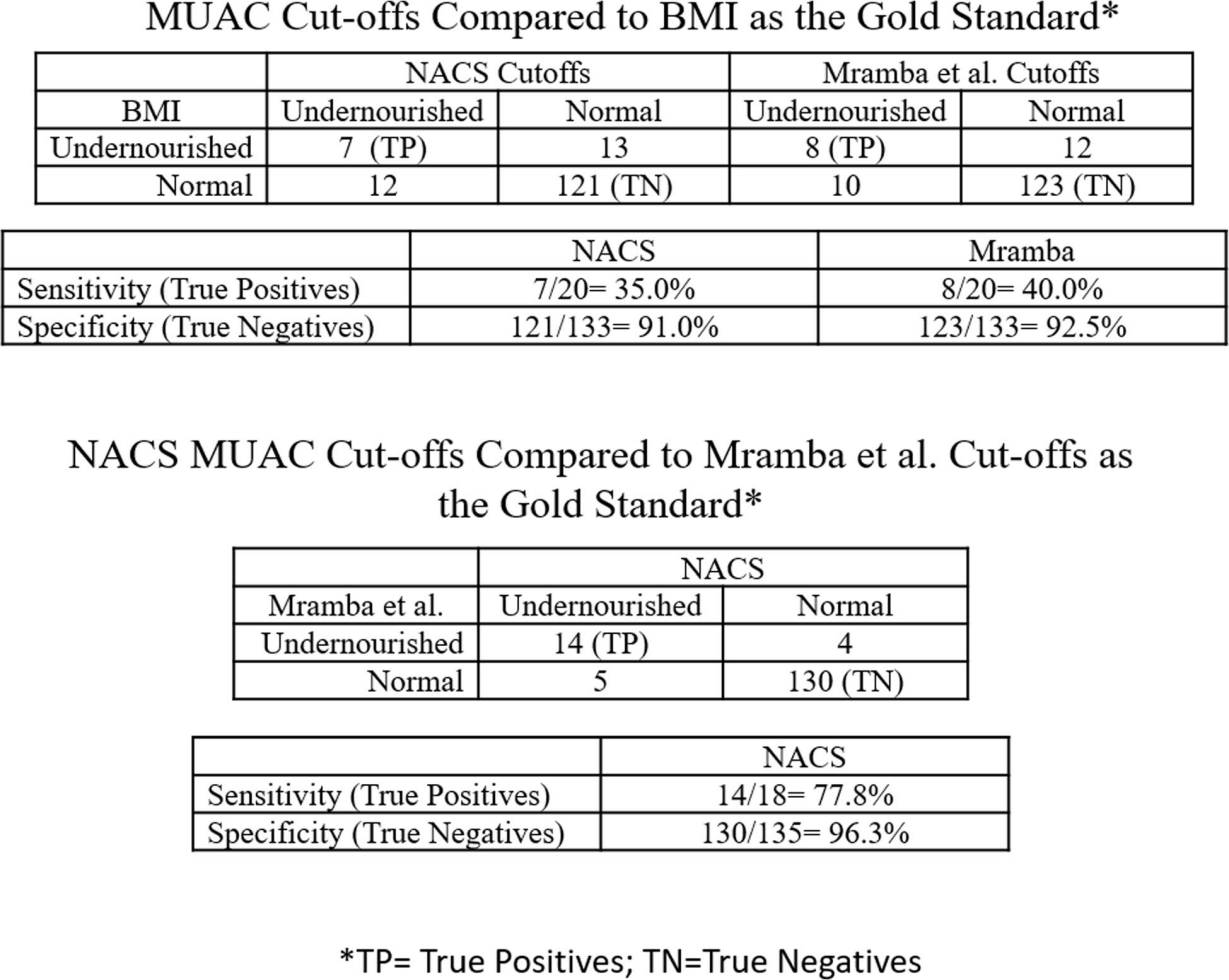
Cross Tabulations of Available MUAC Cut-offs

Previous literature assessing the use of MUAC in adolescents has used BMI as a standard comparison [17, 18, 20, 34]. Sensitivity and specificity were calculated for the *NACS* and Mramba et al. cut-offs when compared to BMI-for-age z-score cut-offs. Both MUAC cut-offs had low sensitivity (*NACS*: 35.0%; Mramba et al.:40.0%) and high specificity (*NACS*: 90.1%; Mramba: 92.5.3%) (Fig. 2). Additionally, if we are to compare the NACS MUAC cut-offs to the Mramba et al. cut-offs as a gold standard, NACS has 77% sensitivity and 96% specificity (Fig. 2).

## Discussion

The data generated from the pilot study for Adolescent Wellness Visits offered an important opportunity to use secondary data to address the data gap in descriptive information about adolescent nutrition in Tanzania and to add to the continued inquiry of using MUAC in adolescent populations. Studies solely created to assess nutritional status, studies with larger sample sizes, and more population-level data collection are needed to confirm our findings and to make country level decisions about programing and policies concerning adolescent nutrition and MUAC cut-offs.

### Nutritional status of young school-age adolescents in Tanzania

About one-quarter of our study participants were outside normal nutritional parameters based on BMI. Our adolescent study population was both under- and over-nourished, while this result may not be generalizable to the Tanzanian adolescent population it offers an opportunity to consider how interventions and programming could dually address under- and overnutrition among adolescents.. Results also indicate that adolescent males and females may have different and distinct nutritional needs. We found that among this sample of Tanzanian adolescents there was significantly more stunting in males than in females. Stunting offers important information of the health status of the population considering that stunting is the result of, in part, poor maternal health and chronic infection [8, 9]. Interpretation of the ten-fold difference in stunting status should be tempered by acknowledging the sample size and the need for more population based data from adolescent populations to confirm this finding. That said, this sex difference clearly aligns with the literature. A systematic review by Wamani et al., of DHS data from 10 sub-Saharan African countries, found that male children under-five have consistently higher rates of stunting than their female counterparts when considering both mean height-for age z-score and stunting prevalence [35]. Male participants in our sample also had higher rates of undernutrition. This aligned with trends showing differences in undernutrition between males and females in East Africa with more males ages 15–19 being underweight than their female counterparts (Uganda: 12.6% females, 26.3% males; Rwanda: 10.9% females, 30% males) [36, 37]. Both higher susceptibility to stunting and undernutrition in males is further confirmed in a systematic review by Akombi et al., determining the factors associated with stunting, wasting and underweight in Sub-Saharan Africa, which found that most studies in their review reported higher susceptibility to stunting, wasting, and underweight in male children than female children [38]. The results also highlight that the young adolescent females in our sample had higher rates of overnutrition than males. This follows the same trend as reported in other recent DHS data in East Africa with females ages 15–19 having higher prevalence of overweight or obesity than males ages 15–19 (Uganda: 11% females, 1% males; Rwanda: 13% females, 1% males) [36, 37]. While the Tanzania DHS does not report nutritional status for men, the percentage of women ages 15–19 in Tanzania who are overweight or obese has increased from 11% in the 1991–1992 survey to 28% in the 2015–2016 survey [26]. With clear data of under- and overnutrition in middle and later adolescences (ages 15–19), plus indications of both under- and overnutrition during younger adolescence (~ages 12–15), there is a distinct need for prevention efforts to start as early as possible to circumvent poor nutritional status continuing into adulthood. Adolescent specific nutrition programs could offer necessary sources of positive mental and physical growth during an important time in development especially when adolescents are at higher risk for adverse health outcomes due to stunting, undernutrition, and overnutrition [9].

### Relationship between MUAC and BMI

Aligning with previous literature, the results of this study indicate that BMI and MUAC measurements are positively correlated for young adolescents [17–23]. Our finding are the first to compare the BMI and MUAC relationships stratified by sex and to show that the correlation between BMI and MUAC is weaker in males. Further studies replicating this finding are needed to better understand the cause of the weaker correlation and if this is due to random chance, true differences between males and females, or to systematic error such as outliers of BMI and MUAC in the male study sample, that boys are misclassified due to stunting, or due to measurement errors [39, 40].

Because MUAC and BMI show a stronger relationship in females who are overnourished than those who are undernourished, this indicates a need to better understand the use of MUAC in populations susceptible to overnutrition. There are some studies with evidence to support the use of MUAC to identify overweight and obese populations of adolescents [21, 41, 42], so it will be important to further consider the relationship between MUAC and BMI in future research to understand whether it is feasible to include cut-offs for overweight in existing MUAC cut-offs.

Based on our exploratory sub-group analysis of comparing BMI and MUAC by self-reported pubertal status in males and females, there is no evidence from the current study to suggest that pubertal status affects the BMI and MUAC relationship. Previous studies assessing the BMI and MUAC relationship in adolescents have not stratified the analysis by pubertal status [17, 18, 20, 21]. Body proportions and fat distributions change rapidly during puberty; males gain more muscle mass in their trunk and upper body, while females gain more fat mass in their hips and thighs [43]. While on a population level, the effects of puberty may be normalized through age and sex standardized z-scores, it will be important to further consider how puberty may affect screening of individual children. In LMICs it will be prudent to understand whether BMI or MUAC is more or less valid when measuring the nutritional status of an adolescent pre- or post-puberty and whether it is more or less valid for boy or girls at different stages in puberty.

### Currently available MUAC and BMI cut-offs

While we identified adolescents outside the normal nutritional ranges, the sensitivity and specificity results showed that there was inconsistency between the different cut-offs (WHO’s BMI-for-age z-score cut-offs, cut-offs provided by the NACS materials, and Mramba et al.’s newly forms MUAC-for-age z-score cut-offs). Because the sensitivity of both MUAC cut-offs was low compared to BMI for age cut offs, we must consider what this means for the practical use of these measurements. For example, when using BMI and MUAC concurrently, which cut-off should prompt a referral? Because the NACS and the Mramba et al. cut-offs do not align, it will be important to also further consider the practical use of the Mramba et al. z-scores and how they can inform country-level MUAC cut-offs like the ones found in the NACS job aids and training manuals. For example, the Mramba et al. cut-offs could help inform sex-specific cut-offs for future NACS tools which currently do not have sex-specific cut-offs. The potential value of the MUAC measurement is limited to undernutrition because current cut-offs only assess undernutrition and not overnutrition. The available MUAC cut-offs only categorize those who are undernourished and do not provide cut-offs for those who are over nourished, representing a distinct complementary need for BMI as a common nutritional indicator and, again, a better understanding of MUAC and BMI in adolescents who are in non-normal categories of nutritional cut-offs. However, in fragile or vulnerable settings where high undernutrition is likely, the MUAC could be valuable tool for quick adolescent assessments.

### Study strengths and limitations

This was the first analysis to examine the relationship between BMI and MUAC in adolescents in Tanzania, a setting with limited information on young adolescent nutrition. Most nutrition information about adolescents in LMICs is taken at the clinic level, often in populations with HIV [4, 5]. This study is unique in that it was collected in collaboration between schools and clinics among students of varying health and nutritional status. This study also had several limitations. First, due to the small sample size and the narrow scope of this study to two schools in Tanzania, generalizability among adolescents across Tanzania is limited. Second, while BMI is often used as the standard comparison to MUAC, this was the first time the participating clinicians were calculating BMI with BMI wheels and measuring MUAC on adolescents. Height, weight, and MUAC are all measurements that are prone to measurement error due to inter-observer differences in measurement or rounding errors [44]. Additionally, since this was a pilot study using secondary data and given the limitations of our measures of puberty, in future studies it will be important include more robust measures of puberty in population-based nutrition data to better understand the effect of puberty on the BMI and MUAC relationship. Alternative indicators for puberty may include multiple self-reported questions about various stages of puberty or a physical exam by a clinician [44].

### Research recommendations

Future population-based studies need to be done with more nutritionally and socially diverse populations in Tanzania and globally, to be able to better understand the nutritional status of adolescents and to contribute data for cut-off recommendations. For example, by adding children and adolescents ages 5–15 in a subsample of the DHS with indicators like BMI and MUAC, Tanzania and other LMICs will be better equipped to track and understand the nutritional status of their adolescents. Larger and more diverse sample sizes will contribute to informing sex-specific cut-offs for MUAC in further research. The relative contribution of using the MUAC as an indicator of overnutrition requires additional research as does how stunting affects under and overnutrition measurements.

### Program and policy recommendations

The high prevalence of stunting in this population of adolescent males indicates a distinct need to target cycles of undernutrition and chronic infection that contribute to stunting. This requires continuing to target mothers and children under five, but it also requires extending the age range of “child” services so that children above 5 years old also receive necessary and affordable nutrition support services. During a study dissemination meeting with Tanzanian stakeholders about the larger study of wellness visits held in October 2018, parents and school staff were acutely aware of the nutritional issues for young adolescents and were eager to seek information and guidance from health providers about how to improve the nutritional status of adolescents. This awareness was articulated with an acknowledgement that nutritional status and educational outcomes are closely linked and both the health and education sectors can work together to support this agenda.

More pragmatically, within health services, it would still be optimal for both the MUAC and BMI measures to be taken for adolescent assessments particularly because as indicated in global guidelines, the MUAC is accurate in cases where BMI is not a proper measurement due to conditions affecting one’s weight such as adolescents who are pregnant or have HIV. In addition, in the absence of the equipment or training to properly calculate BMI, the MUAC could offer a feasible and informative alternative although additional research on adolescent cut-offs would strengthen this recommendation.

## Conclusion

This study contributes to the literature on adolescent nutrition in a low-resource setting and to the evidence base on MUAC assessments as an indicator for nutritional status in adolescents. Given that BMI and MUAC are correlated in this population of adolescents, MUAC may offer a promising method for simple, low resource nutrition screening when BMI is not possible. More studies are needed in adolescents who are identified as having non-normal nutritional status to understand the relationship between BMI and MUAC on a more nuanced level.

## Data Availability

The datasets generated and/or analyzed during the current study are not publicly available but are available from the corresponding author on reasonable request.

## Abbreviations

BMI: Body Mass index
DHS: Demographic and Health Service
LMIC: Low and Middle Income Countries
MUAC: Mid-Upper Arm Circumference
NACS: Tanzania Nutrition Assessment, Counseling, and Support
